# Poster Session II - A221 INVESTIGATING INFLAMMATORY BOWEL DISEASE (IBD) THROUGH THE LENS OF AUTOPHAGY AND MITOCHONDRIA

**DOI:** 10.1093/jcag/gwaf042.221

**Published:** 2026-02-13

**Authors:** A Jaiswal, S Krishnan, A Wang, A Mohan, D McKay, T Shutt

**Affiliations:** Biochemistry and Molecular Biology, University of Calgary Cumming School of Medicine, Calgary, AB, Canada; Biochemistry and Molecular Biology, University of Calgary Cumming School of Medicine, Calgary, AB, Canada; Biochemistry and Molecular Biology, University of Calgary Cumming School of Medicine, Calgary, AB, Canada; Biochemistry and Molecular Biology, University of Calgary Cumming School of Medicine, Calgary, AB, Canada; Biochemistry and Molecular Biology, University of Calgary Cumming School of Medicine, Calgary, AB, Canada; Biochemistry and Molecular Biology, University of Calgary Cumming School of Medicine, Calgary, AB, Canada

## Abstract

**Background:**

Substantial data demonstrates perturbation of epithelial cell mitochondrial form and function in both *in vitro* and animal models of colitis, as well as in tissue samples from individuals with IBD. The T300A loss of function mutation in the autophagy gene ATG16L1 is the second most frequent variant observed in individuals with IBD. Yet, the effect of IBD genetic susceptibility traits on mitochondrial function is poorly understood. We test the hypothesis that the ATG16L1 T300A variant causes dysregulated mitochondrial autophagy, loss of mitochondrial function, and the release of pro-inflammatory mitochondrial DNA (mtDNA) into the cytosol.

**Aims:**

1. To determine if ATG16L1 deficiency disrupts epithelial mitochondrial function in the context of IBD.

2. To examine whether ATG16L1-T300A variant alters epithelial mitochondrial function in the context of IBD.

**Methods:**

ATG16L1 knockout (KO) epithelial cell lines (HEK293T and HCT116) were assessed for general autophagy and mitophagy using the Mito-QC fluorescent reporter. Mitochondrial mass was measured by flow cytometry, mitochondrial oxygen consumption was evaluated using the Resipher platform, and mtDNA release was quantified by image-based analysis. Wild-type (WT) and T300A variant forms of ATG16L1 were re-expressed in ATG16L1 KO cell lines, and assessed as above for the ability rescue KO phenotypes.

**Results:**

Consistent with defective mitochondrial turnover, mitophagy was significantly reduced in both HEK and HCT116 ATG16L1-KO cell lines, which also exhibited more elongated and fused mitochondrial networks. This result was accompanied by a ∼27% increase in mitochondrial mass compared to wild type HEK cells (n = 4 biological replicates; p = 0.0236). Indicative of reduced oxidative phosphorylation, ATG16L1-KO HEK cells had a 50% reduction in basal oxygen consumption rate (OCR) (n = 2 biological replicates; 3 technical replicates; p = 0.0006). Moreover, mtDNA release was increased by ∼2.5-fold in ATG16L1-KO HEK cells, suggesting enhanced pro-inflammatory signal (n = 2; 10 images per replicate; p < 0.0001). Similar mitochondrial defects were observed in HCT116 cells. Re-expression of wild-type ATG16L, but not the T300A IBD risk variant, rescued these defects, restoring mitophagy, mitochondrial network morphology, and OCR to levels in wild type cells.

**Conclusions:**

These proof-of-concept studies suggest that an individual with IBD harbouring the T300A ATG16L1 mutation may have significant loss of mitochondrial function, which would be predicted to result in reduced ATP production and consequently loss of barrier function. These data highlight an underexplored role of mitochondrial stress in ATG16L1-related IBD pathogenesis, and the potential for targeted therapies to restore mitochondrial homeostasis in enteric inflammation.

**Funding Agencies:**

CIHR

